# Improved functionality of *Ligilactobacillus salivarius* Li01 in alleviating colonic inflammation by layer-by-layer microencapsulation

**DOI:** 10.1038/s41522-021-00228-1

**Published:** 2021-07-09

**Authors:** Mingfei Yao, Yanmeng Lu, Ting Zhang, Jiaojiao Xie, Shengyi Han, Shuobo Zhang, Yiqiu Fei, Zongxin Ling, Jingjing Wu, Yue Hu, Shouling Ji, Hao Chen, Björn Berglund, Lanjuan Li

**Affiliations:** 1 State Key Laboratory for Diagnosis and Treatment of Infectious Diseases, Collaborative Innovation Center for Diagnosis and Treatment of Infectious Diseases, National Clinical Research Center for Infectious Diseases, The First Affiliated Hospital, College of Medicine, Zhejiang University, Hangzhou, China; 2 Department of Bone marrow, The First Affiliated Hospital, College of Medicine, Zhejiang University, Hangzhou, China; 3 College of Computer Science and Technology, Zhejiang University, Hangzhou, China; 4 Center for molecular Imaging Research, Shanghai Institute of Materia Medica, Chinese Academy of Sciences, Shanghai, China; 5 Department of Biomedical and Clinical Sciences, Linköping University, Linköping, Sweden

**Keywords:** Applied microbiology, Microbiome

## Abstract

The low viability during gastrointestinal transit and poor mucoadhesion considerably limits the effectiveness of *Ligilactobacillus salivarius* Li01 (Li01) in regulating gut microbiota and alleviating inflammatory bowel disease (IBD). In this study, a delivery system was designed through layer-by-layer (LbL) encapsulating a single Li01cell with chitosan and alginate. The layers were strengthened by cross-linking to form a firm and mucoadhesive shell (~10 nm thickness) covering the bacterial cell. The LbL Li01 displayed improved viability under simulated gastrointestinal conditions and mucoadhesive function. Almost no cells could be detected among the free Li01 after 2 h incubation in digestive fluids, while for LbL Li01, the total reduction was around 3 log CFU/mL and the viable number of cells remained above 6 log CFU/mL. Besides, a 5-fold increase in the value of rupture length and a two-fold increase in the number of peaks were found in the (bacteria-mucin) adhesion curves of LbL Li01, compared to those of free Li01. Oral administration with LbL Li01 on colitis mice facilitated intestinal barrier recovery and restoration of the gut microbiota. The improved functionality of Li01 by LbL encapsulation could increase the potential for the probiotic to be used in clinical applications to treat IBD; this should be explored in future studies.

## Introduction

Inflammatory bowel disease (IBD) constitutes an emerging set of diseases, with increasing incidence and prevalence worldwide^1^. IBD includes ulcerative colitis and Crohn’s disease, which are chronic, life-long, and relapsing diseases of the gastrointestinal (GI) tract caused by a range of genetic and environmental factors^2^. IBD still remains incurable despite many clinically available therapeutic interventions, and the exact mechanism of IBD development remains unknown. Recent studies have found dysregulated interactions among the intestinal bacteria, the gut barrier, and the intestinal-associated immune system in patients with IBD. Typically, the diversity of the gut microbiota and the relative abundance of *Firmicutes*, *Bacteroidetes* and *Actinobacteria* has been found to be decreased in patients with IBD^2,3^. Therefore, the gut microbiome is considered a potential, novel target for IBD treatment^4,5^.

Orally administered probiotics show considerable potential as an alternative treatment of IBD due to their ability to reestablish gut microbiota homeostasis, restore gut barrier integrity, modulate immune responses, protect against invading pathogens and prevent chronic inflammation^6^. *Lactobacillus* and *Bifidobacterium* probiotics, such as *Bifidobacterium longum*, *Lactobacillus acidophilus*, and *Ligilactobacillus salivarius*, have been confirmed to exert anti-inflammatory activities in multiple in vitro and in vivo studies, by increasing the level of anti-inflammatory cytokines while reducing production of inflammatory cytokines such as TNF-α, IL-6, and IL-1β^7–11^. *Ligilactobacillus salivarius* Li01, isolated from feces of healthy individuals, has been proven to be able to protect the intestinal barrier^12^, decrease the serum levels of inflammatory cytokines and bacterial translocations, and enhance the abundance of the gut microbiota^13^. These abilities indicate that administration of Li01 has great potential in preventing or alleviating colonic inflammation through modulation of the gut microbiota.

For probiotics to function in the intestine, they need to remain viable during storage and gastrointestinal transit; however, Li01 is very susceptible to environmental factors such as oxygen, gastric acid, and bile salt. Moreover, therapeutic efficacy may require promotion of colonization as the probiotics may also face colonization resistance from commensal bacteria^14^.

Natural polysaccharides, such as chitosan, pectin, and alginate, are favored in the development of macro/nano formulations to protect active cargo from harsh conditions in the GI tract for the treatment of IBD^15^. Natural cationic polysaccharides exhibit excellent cytocompatibility with no detectable cytotoxicity^16^. Besides, many studies have indicated that polysaccharides can be fermented in gut microbiota and exert prebiotic properties and health-promoting effects^17–19^. For example, alginate increases the abundance of butyrate producing bacteria, including *Bacteroides, Bifidobacterium*, and *Lactobacillus* species^20^. Oral administration of chitosan has been associated with manipulating gut microbiota and ameliorating DSS-induced ulcerative colitis mice^21^. Thus, delivery systems combining probiotics and polysaccharides are actually synbiotics. However, some polysaccharides like carrageenan can also be fermented, their use is limited due to its exacerbation of colitis^15^. The mucoadhesive properties and toxicity of chitosan and alginate have been previously studied. Chitosan has been shown to be highly adhesive to mucin, accompanied by lower biocompatibility but no dramatic effect. Alginate has high mucosal biocompatibility and overall show moderate mucoadhesive properties^22^.

We previously developed microgels with alginate and gelatin complexes as wall materials to encapsulate Li01 cells and protect them from environmental stress^23^. They can significantly improve the stress resistance of Li01 in gastric and intestinal fluids compared to free bacterial cells. One big problem is that the size of microgel particle is usually hundreds of microns, which decreases their retention time and influence the mucoadhesion of probiotics in the intestine. Also, large particle sizes limit their further application in functional food or pharmaceutical industry.

The layer-by-layer (LbL) assembly technique could be utilized to encapsulate probiotics and produce homogeneous nano coatings with precise control of the structure. Moreover, the whole assembly process can be conducted under mild conditions^24^. Thin-wall microcapsules can be formed via LbL self-assembly of oppositely charged polyelectrolytes (alginate and chitosan) on the surface of the cells, which is promising not only for improving the viability of probiotics, but also for facilitating the mucoadhesive function on the intestinal surfaces. More importantly, thin-wall can be strengthened through cross-linking of the alginate molecules with calcium and zinc ions combination^25^, which has not been applied in the encapsulation of probiotics before.

Dextran sulfate sodium (DSS)-induced acute colitis mouse models are widely used for studying clinical and histological features of human ulcerative colitis, colonic inflammation, damaged epithelium barrier and dysregulated host innate immunity and the gut microbiota^26^. The purpose of this study was to evaluate the effects of treatment with LbL encapsulated Li01 (LbL Li01) with a DSS-induced colitis mouse model and compare to treatment with non-encapsulated Li01 (free Li01). Furthermore, the effect of LbL Li01 on the gut microbiota composition in mice was investigated and the mucoadhesive properties of LbL Li01 were evaluated with atomic force microscopy (AFM).

## Results and discussion

### Synthesis and characterization of Li01-loaded layer-by-layer systems

**Fig. 1:**
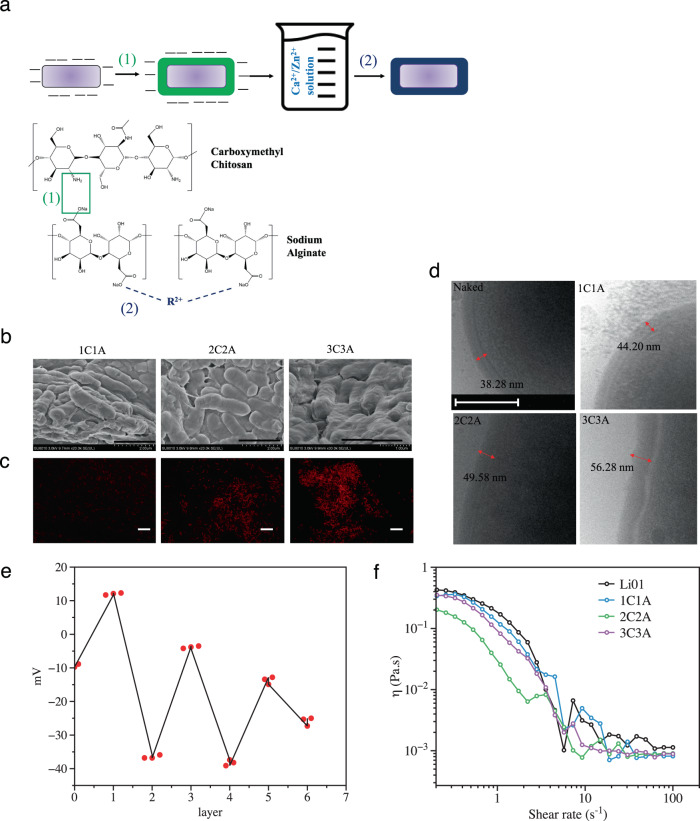
Characterization of LbL Li01. Preparation progress of LbL Li01 and was shown in (**a**). Step (1) shows the molecular reactions between alginate and chitosan to form bilayer; step (2) shows cross-linking formed between alginate molecules. Characterization of morphologies of Li01 cells coated with one (1C1A), two (2C2A), and three (3C3A) bilayers by scanning electron microscopy (SEM, scale bars represent 2.0 μm) (**b**) and confocal microscopy (scale bars represent 50 μm) (**c**). The membrane thickness of Li01 cells and cells coated with 1–3 bilayers were determined by cryo-TEM (scale bars represent 200 nm) (**d**). The ζ-potential was measured when each layer was added (**e**). The apparent viscosity of Li01 cells coated with 1–3 bilayers was measured with the shear rate ranging from 0.2 s^−1^ to 100 s^−1^ (**f**).

The process of LbL encapsulation entails the formation of nano-laminated biopolymer coatings enveloping the probiotic bacteria. In this study, the probiotic strain *L. salivarius* Li01 was LbL encapsulated by decoration with chitosan and alginate alternately. The mucoadhesive properties and toxicity of these two wall materials have been previously studied. Chitosan has been shown to be highly adhesive to mucin, accompanied by a slightly low biocompatibility. Alginate has high mucosal biocompatibility and overall shows moderate mucoadhesive properties^22^. However, in the current study, the ζ-potential of Li01 cells were tested and were found to exhibit negative charges (around −9.24 mV) (Fig. 1). Therefore, the encapsulation process begins with coating a chitosan (positive charge) layer followed by an alginate (negative charge) layer to form one bilayer. Figure 1a shows the schematic diagram of LbL Li01 preparation progress. Since normal chitosan is water insoluble, carboxymethyl chitosan was used in this study. Alginate molecules can be cross-linked to a greater extent with a combination of calcium and zinc ions (R^2+^) compared to calcium ions alone. Except binding to the poly-guluronic acid units of alginate and producing a so-called “egg-box” structure, the R^2+^ solution has been confirmed to bind different sites of the alginate molecule^25^. Therefore, after the alginate-chitosan (AC) bilayers were formed, the shell was reinforced by cross-linking the free carboxyl groups of alginate molecules (Fig. 1a).

Li01 was decorated with different numbers of layers, with the maximum of three chitosan-alginate (CA) bilayers. The morphology and physiochemical properties of LbL Li01 are shown in Fig. 1b–f. When the cells were decorated with only one bilayer, their surfaces were found to be not entirely covered by the biopolymers (Fig. 1b). The surface shell became thicker and more compact after coated with two or three bilayers. The fluorescence intensity of LbL Li01 prepared with rhodamine-conjugated alginate was observed to increase with increasing numbers of bilayers (Fig. 1c), indicating the cells could be coated by multiple CA bilayers. Usually, an individual cell can be coated with ~2–10 layers of biopolymers with a thickness of ~4–5 nm of each bilayer^27^. The thickness can be modulated through adjusting processing parameters such coating material concentrations and ionic strength^24^. In the current study, the thickness of the coated envelope of Li01 was determined by cryo-TEM observation. The surfaces became rougher after coated with bilayers and the thickness of the coated envelope increased by 5–7 nm when one more bilayer was added (Fig. 1d). The thickness of the membrane of a bacterial cell changed from 38 to 56 nm after three bilayers were decorated on the surface.

The ζ-potential of Li01 changed dramatically during the LbL preparation process (Fig. 1e). The ζ-potential shifted from positive to negative when chitosan and alginate were coated alternately, indicating that polyelectrolytes were successfully attached on the surface of the probiotics. However, as the number of layers increased, the magnitude of the change in ζ-potential became less pronounced. Polyelectrolyte complexes were formed through electrostatic interactions between the amino groups in the chitosan molecule and the carboxyl groups in the alginate molecules. It can be inferred that before all the carboxyl groups on the alginate layer get saturated by amino groups, the space surrounding the outer layer is already occupied by chitosan molecules.

Increasing the viscosity of the delivery system may affect the gastrointestinal transit of ingested foods, especially by prolonging the retention time^28,29^. Clearly, decreasing the transportation time in the upper GI tract should improve the viability of probiotics. At lower shear rates, the viscosity of the LbL Li01 decreased after coated with bilayers (1C1A, 2C2A, & 3C3A) (Fig. 1f). When Li01 coated with two bilayers (2C2A), it showed the lowest viscosity.

The higher viscosity of non-encapsulated Li01 bacteria may be related with the production of exopolysaccharides (EPS) of Li01^30^. The EPS fail to diffuse out after probiotic were encapsulated, so that the viscosity of 2C2A was much lower. The increase viscosity of 3C3A may be caused by the coated alginate of the out layer. Since particle size were increased after layer-by-layer encapsulation, higher amount of alginate attached on the out layer of LbL Li01^31^. Therefore, in this study, 2C2A was considered as the optimal coating scheme and was used in the subsequent experiments.

### Viability and mucoadhesion of Li01

**Fig. 2:**
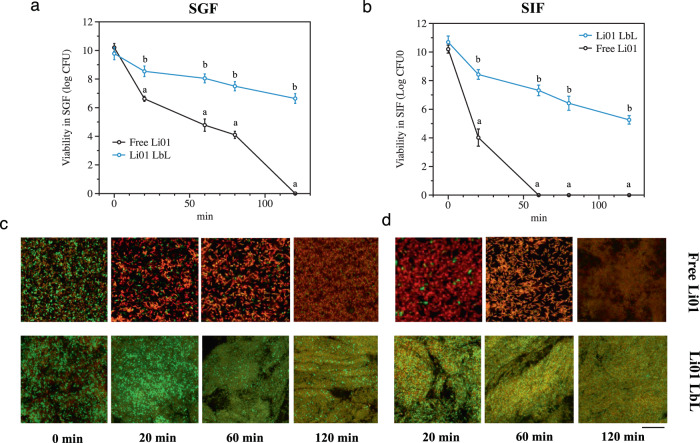
Viability of probiotics during GI transit. Viability count of non-encapsulated (Free) Li01 and LbL Li01 in simulated gastric fluid (SGF) (**a**) and simulated intestinal fluid (SIF) (**b**) after incubation for 0, 20, 60, 80, and 120 min. A small portion of bacterial samples in both SGF (**c**) and SIF (**d**) collected at the time 0, 20, 60, and 120 min in both were stained and visualized by using confocal microscopy (**c**, **d**). Scale bar at lower right represents 50 μm. Data are presented by mean ± SD, *n* = 6, different letters indicate significant different between groups (P < 0.05).

After oral administration, probiotics pass through the upper GI tract before they reach their functional site of activity in the intestine or colon. The survivability of free Li01 and LbL Li01 to simulated gastric fluids (SGF) and simulated intestinal fluids (SIF) was quantified by a plate count method and a LIVE/DEAD *Bac*Light bacterial viability kit to determine its ratio of viable to dead bacteria. Li01 cells are more susceptible to bile salts compared to gastric acids (Fig. 2a–d). Bile primarily exerts its antimicrobial effects on cell membranes and by disturbing macromolecule stability, including on DNA and RNA^32^. Enhanced resistance in SGF was observed in encapsulated Li01 (Fig. 2a, c); after incubation for 20 min, viable free Li01 decreased from 10.2 log CFU/mL to 6.3 log CFU/mL compared to a decrease of 1.2 log CFU/mL among LbL Li01. In SIF, the number of viable, free Li01 cells were quickly reduced from 10.2 log CFU/mL to 4.0 log CFU/mL in 20 min (Fig. 2b, d). With confocal microscopy, most free Li01 cells were found to be dead in SGF and SIF whereas most of the LbL encapsulated cells appeared to be viable (Fig. 2c, d). After incubation for 2 h, no viable cells could be detected among the free Li01 either with viable count or microscopy. However, for LbL Li01, the total reduction was 3 log CFU/mL and the viable number of cells remained above 6 log CFU/mL. Besides, swelling properties of CA bilayers were different as shown in Fig. 2c, d, which was found increased in SIF than in SGF. This may be attributed to protonation and deprotonation of carboxylic acids of alginate at different pH conditions. In SGF, carboxylic acids undergo pronation so that the swelling of crosslinked alginate was quite limited. While at neutral conditions, carboxylic acids were deprotonated, leading to higher swelling of the alginate gels^33,34^. Due to the favorable swelling properties of CA bilayers may facilitate permeating the harsh compounds and provided good protection for cells^12^.

**Fig. 3:**
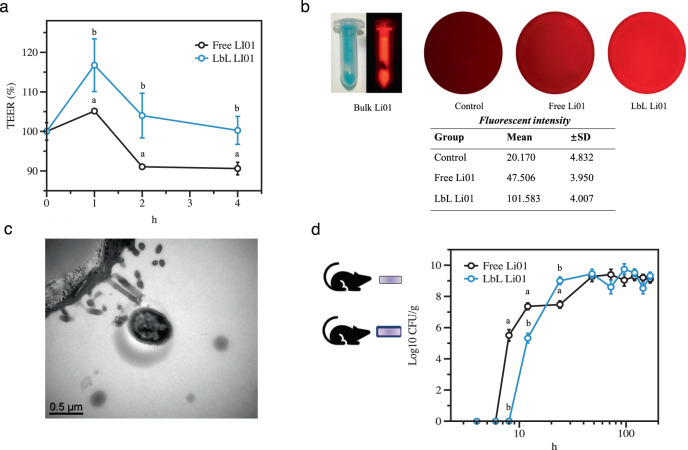
Adhesion of free Li01 and LbL Li01 on a Caco-2 cell monolayer model. The effect of probiotics on transepithelial electrical resistance (TEER) is shown in (**a**). Li01 adhesion on the cell monolayer was visualized by an in vivo imaging system (IVIS) and the fluorescence intensity was quantified. Fluorescence intensities are presented as means from triplicates ± standard deviation (SD) (**b**). Li01 cell attachment to the monolayer was visualized by TEM imaging (**c**). The concentrations of probiotics were monitored in the feces of germ-free rats during one week after oral administration of free Li01 or LbL Li01 (**d**). Data are presented by mean ± SD, *n* = 5, different letters indicate significant differences between groups (*P* <0.05).

Although Caco-2 cell monolayer model is not a perfect model for evaluating probiotic adhesion on intestinal epithelium, it is widely accepted and frequently used^35,36^. Herein, the impact of LbL encapsulation on probiotic adhesion was assessed on this model. Besides, the transepithelial electrical resistance (TEER), as an indicator of the integrity of epithelium barrier, was also measured (Fig. 3a). After being exposed to either non-encapsulated Li01 or LbL Li01 for 1 h, the TEER of Caco-2 cell monolayers was observed to increase. Compared to cells exposed to non-encapsulated Li01, the increased TEER levels of monolayers treated with LbL Li01 were significantly higher; 16.75 ± 6.66% *versus* 5.14 ± 0% (P < 0.05). The amount of bacteria adhered on the Caco-2 cell monolayer was measured as shown in Fig. 3b. As was expected, the fluorescent intensity was doubled, indicating a higher number of LbL Li01 cells adhere to the monolayer as compared to non-encapsulated Li01 cells. Figure 3c displayed the microstructures of the bacteria attached to the monolayers, which confirmed that Li01 cells attached to the microvilli of the Caco-2 monolayer.

To determine the dynamics of probiotic GI transit and colonization in vivo, a germ-free rat model was used. The rats were fed through gavage with free Li01 or LbL Li01. As shown in Fig. 3d, among rats fed with free Li01, the probiotics were first detectable in the feces 8 h after oral administration, whereas probiotics were detectable first after 12 h in feces from rats fed with LbL Li01. Since LbL Li01 exhibits better mucoadhesive effect, their retention time in the intestine was likely longer than free Li01. Among rats fed with LbL Li01, the fecal concentration of Li01 quickly increased to 9 log CFU/g after 24 h. This level remained stable with only mild fluctuations during the following days of observation. Among rats fed with free Li01, the fecal concentration of Li01 increased to 7 log CFU/g after 12 h but similarly reached a level of ~9 log CFU/g 48 h after gavage. These results also indicate that LbL Li01 seems more efficient in colonizing the intestine compared to free Li01 cells.

**Fig. 4:**
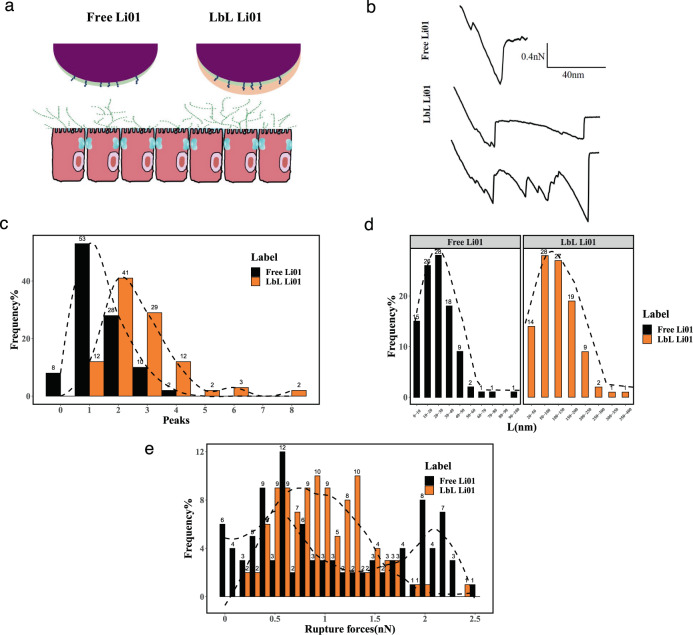
Strength and dynamics of the interactions between mucin and free Li01 and LbL Li01. **a** Li01-mucin and LbL Li01-mucin interactions were determined by measuring the binding forces between a mucin tip and Li01/LbL Li01 by using AFM. **b** Representative force curves of Li01-mucin and LbL Li01-mucin. Number of ruptures histogram (**c**), rupture length histogram (**d**) and adhesion force histogram (**e**) were obtained by recording force curves in a buffer between a mucin tip and Li01. The black line is a Gaussian fit to the data. Each data point in this plot represents the mean ± SEM (*n* = 100 force curves).

Research has demonstrated that alginate and chitosan have mucoadhesive properties and can adhere to the mucus to increase residence time, thus facilitating colonization of probiotics in the intestine^37^. Mucus is a dense network of macromolecular components and their major constituents are heavily glycosylated mucins^38,39^. Bacterial cell surface constituents such as pili play key roles in promoting adhesive interactions with mucus and epithelial cells^40^. In order to fully understand the effects of LbL encapsulation on the interactions occurring between Li01 and mucins, the adhesion between mucin and Li01 was measured by using atomic force microscopy (AFM) (Fig. 4a). The analysis of the retraction force curves generated during the stretching and withdrawal of the tip from the sample surface provides information on the nature of the association of Li01 and LbL Li01 to mucins^41^. The adhesion peaks observed on the retraction curves were fit to a worm-like-chain model (Fig. 4b), which provide information on the number of rupture events, the rupture length, and the rupture forces^40,41^. As shown in Fig. 4c, the number of rupture events (peaks) between the tip and Li01 is lower than that between the tip and LbL Li01, with an average 1.4 ± 0.8 for the former case and 2.7 ± 1.3 for the latter. As shown in Fig. 4d, a maximal distance of rupture of 22.0 ± 15.6 nm was observed for Li01-mucin *versus* 115.3 ± 68.5 nm for LbL Li01- mucin. Since the surface of Li01 cell was covered with chitosan-alginate bilayers, these biomolecules are stretched during the retraction of the mucin-functionalized AFM. *Lactobacillus spp*. may contain pili or EPS on the surface, which engage in bacterial aggregation and specifically binding mucin^40^. After probiotics were coated with bilayer, the mannuronic content of the alginate, which does not take part in the formation of the ionic gel network, plays a critical role in mucoadhesion by formation of hydrogen bonds and other van der Waals interactions^42^. Research also indicated that the longer contact time. 5 s contact times was able to form up to hundreds bonds, thus more peaks could be present in the adhesion curves^42^. Although no significant difference was found between the average rupture forces of Li01-mucin (1.08 ± 0.75) and LbL Li01-mucin (0.96 ± 0.43), it may be due to the short contact time between the mucin and alginate. While in the body, the contact time can be much longer, the mucoadhesion of LbL Li01 can be greatly enhanced in the body. These results are also in consistent with the colonization of probiotics in germ-free mice.

### Effect of probiotics on alleviating colonic inflammation

**Fig. 5:**
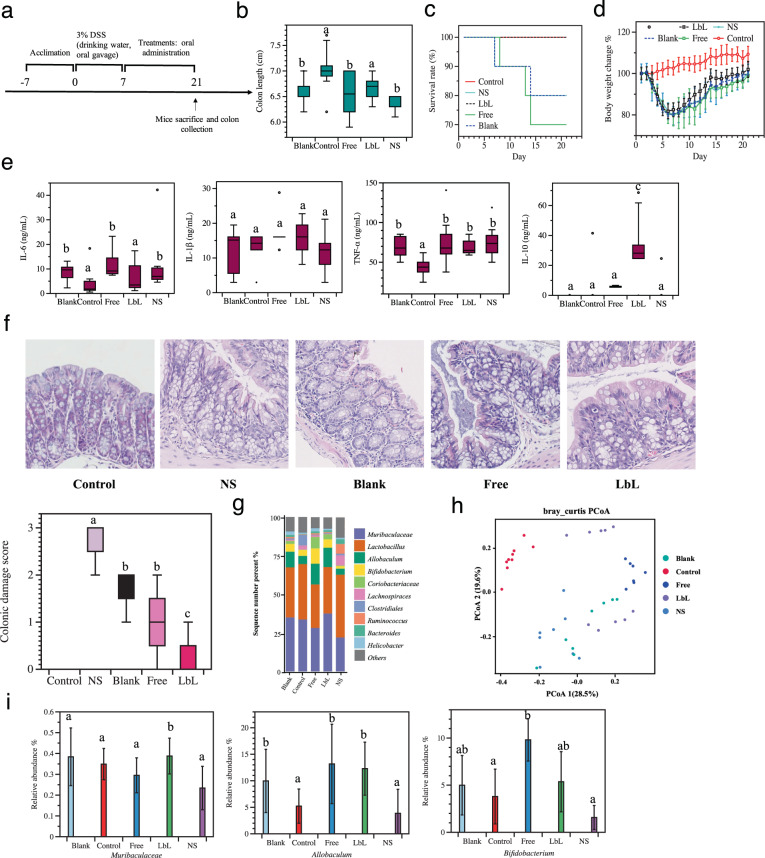
Effects of Li01 treatment of mice with dextran sulfate sodium (DSS)-induced colitis. **a** Chart describing the steps in the DSS-induced colitis mouse model. On the 8th day, mice were stratified on whether they were orally administrated with saline (NS), chitosan and alginate mixture (Blank), non-encapsulated Li01 (Free) or LbL encapsulated Li01 (LbL) for 14 days. **b–d** Colon length, mouse survival rate, and daily body weight changes were measured in each treatment group. (**e**) Concentrations of inflammatory cytokines were measured in the plasma in DSS colitis-induced mice. **f** Images of colonic damage were recorded, and colonic damage scores were measured in DSS colitis-induced mice. **g**, **i** Colonic microbiota profiles and relative abundances of unique microbial taxa following treatment were determined with 16S rRNA-based metagenomic analysis. **h** Microbial clustering is shown based on Bray–Curtis dissimilarity Principal Coordinates Analysis (PCoA) metrics of colic fecal samples of DSS-induced mice after treatment. Data in the figures are presented as mean ± SEM. Different letters represent significant differences (*P* ≤ 0.05) between groups.

The therapeutic efficacy of Li01 and LbL Li01 on colonic inflammation was determined by using a DSS-induced acute colitis mouse model (Fig. 5a–f). The colon length of mice fed with LbL Li01 was significantly longer compared to mice fed with other treatment regimens (Fig. 5b), indicating a superior ameliorative effect of LbL Li01. All mice treated with LbL Li01 were alive at the end of the experiment whereas mice treated with free Li01 had a survival rate of only 70% (Fig. 5c). Moreover, mice treated with LbL Li01 showed a trend of faster recovery of body weight (Fig. 5d).

The levels of pro-inflammatory cytokines (including IL-6, IL-1β, and TNF-α) anti-inflammatory cytokines (IL-10) were also determined in the circulative plasma of the mice after treatment (Fig. 5e). Compared to control group, the level of IL-6 significantly increased in all treatment groups except LbL Li01 group. For IL-1β, all treatment groups exhibited similar levels to the control group, whereas for TNF-α, all treatment groups showed significantly higher levels (*P* < 0.05) compared to the control. Although LbL Li01 showed slightly lower level, no statistically significant differences could be discerned between the treatment groups themselves. LbL Li01 also improved the levels of IL-10 in the plasma compared to the other groups (*P* < 0.05). TNF-α, IL-1β, and IL-6 are produced in response to infections and tissue injuries, and dysregulated, continual synthesis of IL-6 has a pathological effect on colonic inflammation and autoimmunity, whereas expression and secretion of IL-10 can protect from colitis induced by DSS^43^. Our results show that post treatment with LbL Li01 was associated with low levels of the pro-inflammatory cytokines and significant higher levels compared to other groups, suggesting the superiority of LbL Li01 in facilitating colonic epithelial amelioration.

Histological results are consistent with the above results, where significantly lower damage scores in mice treatment with LbL Li01 compared to mice fed with other treatments (P < 0.05), indicating the potential for LbL Li01 to promote rapid recovery of the colonic epithelium (Fig. 5f). The blank group and free Li01 group also exhibited effects in ameliorating colitis to some extent. The function of blank group is due to the prebiotic properties of chitosan and alginate as we described above^19^. Previous study indicated that feeding with killed *Lactobacillus spp*. decrease leaky gut and inflammation and improves physical and cognitive functions, which could explain the slightly enhanced function of free Li01 treatment^44^. Although probiotics belonging to *Lactobacillus* and *Bifidobacterium* seldom induce pro-inflammatory cytokines^45^, studies have demonstrated that both commensal and probiotic bacteria are likely to cause inflammation in the permeable gut, since probiotics could cross a damaged gut barrier and cause an immune response^46,47^. This could explain the levels of the pro-inflammatory cytokine IL-6 observed in mice administered with free Li01. LbL Li01 could potentially be associated with a favorable function due to the mucoadhesive properties of the LbL shells which facilitate Li01 binding to the intestinal mucosal layer.

Inflammatory diseases in the GI tract are always accompanied by dysbiosis of the gut microbiota^48,49^. Oral administration of Li01 may alleviate colitis through modulation of the gut microbiota^12,13^. To examine the effect of free Li01 and LbL Li01 on the composition of the gut microbiota in mice with DSS-induced colitis, a 16 S rRNA gene-based metagenomics was employed. As shown in Fig. 5g, i, compared to mice fed with saline, the relative abundance of *Muribaculaceae, Allobaculum*, and *Bifidobacterium* in mice fed with LbL Li01 was considerably higher. Mice with progressive colitis usually display a depletion of gut commensals in the microbiota like *Muribaculaceae*^50^. *Allobaculum* has been confirmed to produce short-chain fatty acids and to be positively correlated with body weight, which can help maintain colonic function and epithelium structure^51–53^. Compared to free Li01, the abundance of *Muribaculaceae* was higher in LbL Li01 while it did not make any different for *Allobaculum* and *Bifidobacterium*. A high ratio of free Li01 may be killed after ingested by mice. Killed probiotics may also exhibit effects of altering microbiota composition to promote^54^, which may enhance the abundance of *Allobaculum* and *Bifidobacterium*. A Principal Coordinates Analysis (PCoA) of the microbiomes (Fig. 5h) showed the clustering of the control group contra the other experimental groups, expect half of the points in LbL Li01 group, which are very close to the control group. Therefore, treatment with LbL Li01 may facilitate faster restoration of the gut microbiota.

In summary, this work demonstrates the potential of LbL delivery system in enhancing survival and efficacy of *L. salivarius* Li01 cells in the gastrointestinal tract. After encapsulation, their viability in simulated gastrointestinal environment was significantly improved. The other unique feature of the LbL delivery system is its enhanced mucoadhesive properties. These added functions for Li01 cells contributed to their enhanced efficacy of ameliorating DSS-induced colitis in mice. Although the results presented here could indicate a possible application of LbL Li01 in clinical applications for treating IBD, more studies are still required. In future studies, we plan to investigate the function of LbL chronic IBD mouse model. Moreover, a procedure for large-scale production of LbL Li01 will be explored.

## Methods

### Layer-by-layer synthesis and characterization

*L. salivarius* Li01 were cultivated in De Man, Rogosa, Sharpe (MRS) broth (Oxoid, Basingstoke, UK) overnight at 37 °C in an anaerobic chamber (Electrotek Scientific, Shipley, UK), and the bacterial suspension was centrifuged at 4000 rpm for 5 min. After centrifugation, the supernatant was removed and the collected bacterial cells were washed twice with saline buffer and finally, 10^10^ CFU of Li01 were resuspended in 1 ml saline buffer. The Li01 cells were then coated layer-by-layer alternately with alginate (Sigma Aldrich, St. Louis, MO, USA), a anionic polymer, and carboxylation chitosan (Aladdin, Shanghai, China), an cationic polymer, according to a method described previously^37^. Li01 was first incubated within the carboxymethyl chitosan solution (1 mg/mL) at 37 °C for 10 min at 100 rpm in an IKA KS4000i shaker (IKA, Staufen, Germany). The suspension was then centrifuged and washed twice with saline buffer, after which the cells were added to the alginate solution (1 mg/mL) to form a bilayer. This process was repeated until multiple bilayers were formed. The surface potential was measured by using a Zeta-sizer (Malvern Panalytical, Malvern, UK) during the bilayer preparation. The LbL encapsulated bacterial cells were then incubated in a mixed solution (0.05 Mcalcium chloride + 0.05 M zinc sulfate) for 30 min and rinsed with saline buffer. The morphologies of the encapsulated Li01 were characterized by scanning electron microscopy (SEM) (Hitachi SU8010, Tokyo, Japan) and cryo-TEM (Talos F200C 200kv, FEI, USA). In order to evaluate the structure of LbL Li01, Alginate-Rhodamine (Xi’an Ruixi Biological Technology Company, Shanghai, China) was used instead of alginate in the preparation before visualization by a LSM 710 confocal microscope (Zeiss, Oberkochen, Germany). The apparent viscosities of non-encapsulated Li01 and LbL Li01 with 1,2, and 3 bilayers were determined by a HAAKEMARS III rotary rheometer (Thermo Fisher Scientific). Two 60 nm diameter plates with a 1.0 mm set gap between them was used. Samples were measured at shear rates of 0.1–100 s^−1^.

### Viability of encapsulated Li01 and adhesion of bacterial cells on Caco-2 cell monolayers

The viability of LbL Li01 during GI transit was tested with an in vitro digestion model. Simulated gastric fluids (SGF) and simulated intestinal fluids (SIF) were prepared according to a previously described methods with some modifications^23,55,56^. Samples were taken after free Li01 and LbL Li01 were incubated in SGF or SIF for 0, 10, 20, and 40 min separately. A LIVE/DEAD *Bac*Light Bacterial Viability Kit (Thermo Fisher Scientific, Waltham, MA, US) was used according to the manufacturer’s instructions to detect the viability of the cells. Images were taken by using a LSM 710 confocal microscope (Zeiss) with a CCD camera (Nikon, Tokyo, Japan).

Caco-2 cells (passage 40-65) were cultured in complete Dulbecco’s modified essential medium (DMEM) Gibco (Thermo Fisher Scientific, Waltham, MA, USA) containing high concentrations of glucose, 10% ~15% fetal bovine serum, 1% antibiotics, and 1% amino acids, in a 50-mm culture dish. Cells were collected when they reached 70% confluence and seeded at 3 × 10^5^ cells/ml on six-well polyester Transwell plates (Corning Inc., MA, USA)^57^. After culturing for 21 days, Caco-2 monolayers were formed. Cells were fluorescently labeled with vivotag-S750 (PerkinElmer, Waltham, MA, USA) according to the manufacturer’s protocol and were stored in a tube. 1 ml of non-encapsulated Li01 or LbL Li01 were added to the filter of the transwell plates with a concentration of 1 × 10^7^ CFU/ml. Bacteria were removed after 1 h treatment, washed twice with Hank’s balanced salt solution and refilled with DMEM culture medium. The tube and six well plate fluorescent images were taken by the MARS in vivo imaging system developed by Artemis Intelligent Imaging (Shanghai, China), which is equipped with a liquid nitrogen-cooled NIRvana LN InGaAs camera (Teledyne Princeton Instruments, Thousand Oaks, CA, USA), a SWIR lens with 50 mm focal length and a 1000 nm long-pass filter, and shadowless illumination provided by using an 808 nm laser with 90 mW/cm^2^ power density. The transepithelial electrical resistance (TEER) was measured at 0, 1, 2, and 4 h of incubation.

### Bacterial colonization in germ-free rats

The dynamics of bacterial colonization with Li01 in the intestines were investigated in a germ-free SD rat model. Ten male germ-free rats (6–8 weeks old) were maintained according to the methods we established previously^58^. Germ-free rats were fed through gavage with 200 µL of solution containing 10^9^ CFU/mL of non-encapsulated Li01 (*n* = 5) or LbL Li01 (*n* = 5). Three drops of fresh feces were collected at 0, 2, 4, 6, 8, 12, and 24 h, and at 2, 3, 4, 5, 6, and 7 days after gavage and the concentration of Li01 was measured by using a plate count method.

### Ethics

The animal experiments in this manuscript were approved (No.2018755-1) by the Tab of Animal Experimental Ethical Inspection of the First Affiliated Hospital, College of Medicine, Zhejiang University.

### Atomic force microscopy (AFM) analysis

AFM probes (CP-qp-Scont Nanosensors, Watsonville, CA, USA) with borosilicate glass beads (1.5 μm) were functionalized with mucins. Before the mucin-modified tips were prepared, the cantilever spring constant was calibrated by using a thermal K method program equipped with IGOR Pro 6.04 (Wavemetrics, Osewego, OR, USA). The spring constant of the probes was of 0.01 nN/Nm. The tips were first placed in a PSD-UV UV-ozone cleaner (Novascan, Phoenix, AZ, USA) for 8 h, immersed in 1% ethanol solution for 4 h and rinsed with deionized water, then immersed in 10% glutaraldehyde water solution for 2 h and washed with deionized water. Finally, the tips were immersed in a solution with 2 mg/mL mucin from porcine stomach (Sigma Aldrich), which was prepared by adding 200 mg mucin to 100 mL deionized water. After 8 h of incubation, the tips were placed in a PBS solution. Preliminary experiments confirmed that the tip was covered with mucin (data not shown). Tips were rinsed with deionized water before use.

Free Li01 and LbL Li01 bacterial suspension were centrifuged, and the concentration was adjusted to OD (600 nm) value of 0.5. Then 1 mL bacterial suspension was deposited on polylysine modified slides and incubated at 4 °C for 4 h. After the bacterial cells adhered on the slides, they were gently rinsed twice with PBS.

Force measurements were performed at room temperature (25 °C) in saline buffer (pH 7.0) by using an Asylum MFP-3D atomic force microscope (Santa Barbara, CA, USA) with the operation software IGOR Pro 6.04 (Wavemetrics). For each experiment, the force map was recorded on a 5 μm × 5 μm area over the cell surface with a resolution of 16 × 16 corresponding 32 × 32 points (1,024 force curves). All force curves were obtained using a contact time of ~ 2 s, a maximum applied force of 1 nN, and an approach and retraction speed of 400 nm/s. Each group was repeated four times.

### Evaluation of Li01 effect on mice with DSS-induced colitis

Specific pathogen-free C57BL/6 male mice (age = 5 weeks) were purchased from Zhejiang Laboratory Animal Center (Hangzhou, China) and were fed with AIN93G diet and maintained in pathogen-free conditions. After acclimation for one week, mice were randomly assigned to groups and each mouse was labeled in order to record their weight. Mice were randomly assigned to five different groups and treated with different regimens. The weight of each mouse was recorded every day. All mice (except for the mice in the control group) were fed with 3% DSS via the drinking water and fed with 200 μL 3% DSS solution by gavage every day for 7 days. On day 8, feeding with DSS was stopped. Four different treatment regimens were treatment with LbL Li01 (10^9^ CFU/time), free Li01 (10^9^ CFU/time), saline buffer (NS group), and alginate and chitosan mixture (Blank group). After the mice received their treatment regimens for 14 days before they were sacrificed. Blood and colon tissues were collected for analysis of colon inflammation. The length of colon tissue was measured.

### Histopathological analysis

Colon tissues from mice were analyzed in accordance with a previously published protocol^59^. In short, the tissues were excised, distal colonic segments (0.5 cm) were removed and fixed with 4% neutral-buffered formalin (Hepeng Biology, Shanghai, China) for 24 h and subsequently embedded in paraffin. Cross sections of the colon (4 μm) were cut and mounted on slides. The cross sections were then stained with hematoxylin and eosin solution (Sigma-Aldrich). Tissue damage was evaluated by using a score for the severity of epithelial injury, the extent of inflammatory cell infiltration and goblet cell depletion, according to a previously described protocol^59^. Colonic damage was assigned scores as shown in Table 1^26^.

**Table 1:** Colonic damage score criteria used to evaluate the severity of epithelial injury, the extent of inflammatory cell infiltration and goblet cell depletion in mouse tissue.

Score	Colonic damage	Inflammatory cell infiltration	Submucosa	Muscle/serosa
0	Normal	Normal	Normal	Normal
1	Hyperproliferation, irregular crypts, goblet cell loss	Mild	Moderate to severe	Moderate to severe
2	Mild to moderate cypt loss (10–50%)	Modest	Severe	
3	Severe crypt loss (50–90%)	Severe		
4	Complete crypt loss, surface epithelium intact			
5	Small to medium-sized ulcers (<10 crypt widths)			
6	Large ulcers (≥10 crypt widths)			

### Quantification of serum cytokines and chemokines

Serum cytokines and chemokines in the mouse plasma, including interleukin (IL)-1β, IL-6, IL-10, and tumor necrosis factor (TNF)-α were quantified by using a Bio-Plex Pro Mouse Cytokine 23-Plex Panel (Bio-Rad, Hercules, CA, United States), on a MAGPIX system (Luminex, Austin, TX, US) and analyzed by using the Bio-Plex Manager 6.1 software (Bio-Rad) according to the manufacturer’s instructions.

### DNA purification, amplification, and metagenome sequencing

Colonic content was collected and moved to a liquid nitrogen container immediately for temporary storage for about an hour. For long-term storage, the feces samples were immediately transferred to a −80 °C freezer. Bacterial DNA from the feces samples was extracted by using a QIAamp Fast DNA Stool Mini Kit (Qiagen, Hilden, Germany). PCR primers targeting the V3-V4 region of the 16S rRNA gene with specific barcodes were used (338F: 5′-ACTCCTACGGGAGGCAGCA-3′, 806R: 5′-GGACTACHVGGGTWTCTAAT-3′). PCR products were further purified by using the AxyPre DNA Gel Extraction Kit (Axygen Biosciences, Union City, CA, USA) and quantified by using QuantiFluor-ST (Promega, Madison, WI, USA) according to the manufacturers’ instructions. Purified amplicons were pooled in equimolar and paired-end sequenced (2 × 300) on an Illumina MiSeq platform (Illumina, San Diego, CA, USA). Taxonomy-based analyses were performed by classifying each sequence using the Naïve Bayesian Classifier program of the Michigan State University Center for Microbial Ecology Ribosomal Data base Project (RDP) database (http://rdp. cme.msu.edu/) with a 50% bootstrap score. Diversity analysis and taxonomy-based analysis were performed using the methods at 97% similarity level mothur as described in previous research^60^. For clustering analysis on principal coordinate plots, categories were compared and statistical significance of clustering was determined via Permanova.

### Statistical analysis

Figures were generated by using DataGraph (Visual Data Tools, Inc.). Statistically significant was evaluated by one-way ANOVA followed by Tukey’s post hoc test. Nonparametric ANOVA (Kruskal–Wallis test) followed by Dunn’s post hoc test was applied for the data that don’t pass normal distribution test. *P* < 0.05 was considered statistically significant.

### Reporting summary

Further information on research design is available in the Nature Research Reporting Summary linked to this article.

## Supplementary information

Reporting Summary

## Data Availability

16sRNA sequencing data of the metagenome in this study have been deposited in Sequence Read Archive (SRA) with the accession codes from SAMN19349580 to SAMNI19349608. All other data relevant to the article is included in the article. Data are also available from the corresponding author upon request, see author contributions for specific data sets.
