# Supplementary material for: Improved functionality of Ligilactobacillus salivarius Li01 in alleviating colonic inflammation by layer-by-layer microencapsulation
Source: NPJ Biofilms Microbiomes. 2021 Jul 9;7:58. doi: 10.1038/s41522-021-00228-1 (PMC8270932; doi:10.1038/s41522-021-00228-1)
Supplement: Supplementary file 1 — Reporting Summary [file 41522_2021_228_MOESM1_ESM.pdf]

nature research

Corresponding author(s): Lanjuan Li

Last updated by author(s): May 12, 2021

Reporting Summary

Nature Research wishes to improve the reproducibility of the work that we publish. This form provides structure for consistency and transparency in reporting. For further information on Nature Research policies, see our Editorial Policies and the Editorial Policy Checklist.

Statistics

For all statistical analyses, confirm that the following items are present in the figure legend, table legend, main text, or Methods section.

n/a	Confirmed	
☐	☒	The exact sample size (*n*) for each experimental group/condition, given as a discrete number and unit of measurement
☐	☒	A statement on whether measurements were taken from distinct samples or whether the same sample was measured repeatedly
☐	☒	The statistical test(s) used AND whether they are one- or two-sided<br>*Only common tests should be described solely by name; describe more complex techniques in the Methods section.*
☒	☐	A description of all covariates tested
☐	☒	A description of any assumptions or corrections, such as tests of normality and adjustment for multiple comparisons
☐	☒	A full description of the statistical parameters including central tendency (e.g. means) or other basic estimates (e.g. regression coefficient) AND variation (e.g. standard deviation) or associated estimates of uncertainty (e.g. confidence intervals)
☐	☒	For null hypothesis testing, the test statistic (e.g. *F*, *t*, *r*) with confidence intervals, effect sizes, degrees of freedom and *P* value noted<br>*Give P values as exact values whenever suitable.*
☒	☐	For Bayesian analysis, information on the choice of priors and Markov chain Monte Carlo settings
☒	☐	For hierarchical and complex designs, identification of the appropriate level for tests and full reporting of outcomes
☒	☐	Estimates of effect sizes (e.g. Cohen's *d*, Pearson's *r*), indicating how they were calculated

Our web collection on statistics for biologists contains articles on many of the points above.

Software and code

Policy information about availability of computer code

Data collection	Igor Pro Folder, Zen Blue Lite 2.3
Data analysis	Image J V1.50i, Datagraph, Graphpad Prism , R version 3.6.3, Microsoft Excel 365

For manuscripts utilizing custom algorithms or software that are central to the research but not yet described in published literature, software must be made available to editors and reviewers. We strongly encourage code deposition in a community repository (e.g. GitHub). See the Nature Research guidelines for submitting code & software for further information.

Data

Policy information about availability of data

All manuscripts must include a data availability statement. This statement should provide the following information, where applicable:

- Accession codes, unique identifiers, or web links for publicly available datasets
- A list of figures that have associated raw data
- A description of any restrictions on data availability

The datasets generated during and/or analysed during the current study are available from the corresponding author on reasonable request.

Field-specific reporting

Please select the one below that is the best fit for your research. If you are not sure, read the appropriate sections before making your selection.

☒ Life sciences ☐ Behavioural & social sciences ☐ Ecological, evolutionary & environmental sciences

For a reference copy of the document with all sections, see nature.com/documents/nr-reporting-summary-flat.pdf

Life sciences study design

All studies must disclose on these points even when the disclosure is negative.

Sample size	Sample sizes were chosen based on preliminary data demonstrating statistically significant differences for each specific assay.
Data exclusions	No data were excluded from the analyses.
Replication	All experiments were performed with at least three technical replicates on more than one occasion to ensure reproducibility across experiments.
Randomization	Randomization and covariates were not relevant to our study design as we investigated single factors within each study.
Blinding	Blinding was not relevant as all processing methods were done through available software with consistent parameters utilized across all treatment groups.

Reporting for specific materials, systems and methods

We require information from authors about some types of materials, experimental systems and methods used in many studies. Here, indicate whether each material, system or method listed is relevant to your study. If you are not sure if a list item applies to your research, read the appropriate section before selecting a response.

Materials & experimental systems

n/a	Involved in the study	
☒	☐	Antibodies
☐	☒	Eukaryotic cell lines
☒	☐	Palaeontology and archaeology
☐	☒	Animals and other organisms
☒	☐	Human research participants
☒	☐	Clinical data
☒	☐	Dual use research of concern

Methods

n/a	Involved in the study	
☒	☐	ChIP-seq
☒	☐	Flow cytometry
☒	☐	MRI-based neuroimaging

Eukaryotic cell lines

Policy information about cell lines

Cell line source(s)	American Type Culture Collection (ATCC)
Authentication	Cells were purchased from and authenticated by their respective supplier. None of the cells were derived in-house.
Mycoplasma contamination	All cells tested negative for mycoplasma and were expanded in media containing a mycoplasma prophylactic (plasmocin)
Commonly misidentified lines (See ICLAC register)	No commonly misidentified cell lines were utilized in these studies.

Animals and other organisms

Policy information about studies involving animals; ARRIVE guidelines recommended for reporting animal research

Laboratory animals	Specific pathogen-free C57BL/6 male mice (age = 5 weeks) ; Germ-free SD rats
Wild animals	This study did not involve wild animals.
Field-collected samples	Animals were maintained under standard conditions of humidity (50%), temperature (25 ±2 °C) in a 12 h light and 12 h dark cycle. They were fed standard rodent chow and had free access to water, acclimatized for at least one week prior to use. For germ-free rats, three drops of fresh feces were collected at 0, 2, 4, 6, 8, 12, and 24 h, and at 2, 3, 4, 5, 6 and 7 days after gavage

	and the concentration of Li01 was measured by using a plate count method. The rats were sacrificed at Day 7.<br>After the mice received their treatment regimens for 14 days before they were sacrificed. Blood and colon tissues were collected for analysis of colon inflammation. The length of colon tissue was measured.
Ethics oversight	The animal experiments in this manuscript were approved (No.2018755-1) by the Tab of Animal Experimental Ethical Inspection of the First Affiliated Hospital, College of Medicine, Zhejiang University.

Note that full information on the approval of the study protocol must also be provided in the manuscript.
